# Viscous and Failure Mechanisms in Polymer Networks: A Theoretical Micromechanical Approach

**DOI:** 10.3390/ma12101576

**Published:** 2019-05-14

**Authors:** Roberto Brighenti, Federico Artoni, Mattia Pancrazio Cosma

**Affiliations:** Department of Engineering & Architecture, University of Parma, 43121 Parma, Italy; federico.artoni@studenti.unipr.it (F.A.); mattiapancrazio.cosma@unipr.it (M.P.C.)

**Keywords:** polymers, chains network, damage, chains failure, visco-elastic response

## Abstract

Polymeric materials typically present a complex response to mechanical actions; in fact, their behavior is often characterized by viscous time-dependent phenomena due to the network rearrangement and damage induced by chains’ bond scission, chains sliding, chains uncoiling, etc. A simple yet reliable model—possibly formulated on the basis of few physically-based parameters—accounting for the main micro-scale micromechanisms taking place in such a class of materials is required to properly describe their response. In the present paper, we propose a theoretical micromechanical approach rooted in the network’s chains statistics which allows us to account for the time-dependent response and for the chains failure of polymer networks through a micromechanics formulation. The model is up-scaled to the mesoscale level by integrating the main field quantities over the so-called ‘chains configuration space’. After presenting the relevant theory, its reliability is verified through the analysis of some representative tests, and some final considerations are drawn.

## 1. Introduction

Polymers, thanks to their ability to withstand very large deformations prior to failure, in addition to several other useful functionalities, are widely used in various applications including biomechanics, soft actuators, stretchable electronics, and adhesives. However, under mechanical loads, polymers are also prone to failure and show a strain rate dependent response, drawbacks that can limit their applicability. The above mentioned macroscopically observable mechanisms can be conveniently studied and interpreted on the basis of the polymer’s underlying molecular network, made of cross-linked chains whose mechanical behavior is well described by the so-called entropic-based elasticity. In fact, the disorder degree of the network chains can be easily related to the material entropy that, upon stretching, reduces because of the greater ordered conformation assumed by the deformed chains [1,2].

The typical polymer’s underneath network microstructure is shared also by others classes of polymer-like materials, such as biological tissues and natural matters; natural biopolymers, such as DNA, proteins, cellulose, pectin, and soft tissues, as well as synthetic polymers, are obtained via the polymerization of many small molecules, known as monomers, that usually provides the material characteristic physical properties such as high deformability, good toughness, and a viscoelastic response upon mechanical stress. As an example, pectin gel is made by a chains network constituted by segments join together by crystallization to form a three-dimensional network in which water, sugar, and other materials are held [3].

Very promising theoretical models, suitable to describe the mechanics of polymers based on the so-called statistical mechanics approach, have been proposed so far [4,5,6,7,8]. The above-mentioned models can be usefully adopted also to describe the failure of polymers by considering the statistics of the chains scission [9,10,11,12]. Strain stiffening preceded by failure is observed in experiments when the chains are stretched close to their contour length, i.e., in chains elongated such that the end-to-end distance tends to their maximum extension. Collective chains failure corresponds to a macroscale material rupture, such as the necking or crack nucleation phenomena observable at the continuum level.

Among the methods developed to improve the strength and energy dissipation capabilities of polymers, a noticeable enhancement has been observed to be provided by the addition of nanoscale particles (filler), whose main role is to dissipate energy through the matrix-particle debonding mechanism [13]. However, it is worth noting that the occurrence of debonding is not the only failure mechanism that can take place in a nanocomposite polymer: As shown in [14], two modes of failure (i.e., debonding mechanism and cavitation), depending on the matrix-polymer interface strength and the type of filler inclusions, can occur. Higher interfacial adhesion values required, for instance, when high-performance properties against electromechanical stresses are needed, increase the risk of cavities formation, whose presence triggers the appearance of nucleation points of failure [15,16].

Moreover, the rate dependent mechanical response of polymers under mechanical stress also provides a good way to dissipate energy, especially in highly stretched regions such as in the crack tip process zone [17]. Several mechanisms such as chains sliding, reversible bonds and bond exchange, chains rearrangement, and entanglement-disentanglement are responsible for such a rate dependent or viscoelastic response; the wide class of viscoelastic materials includes polymers, biopolymers, metals at high temperatures, and bituminous materials. Their time-dependent response comes from a molecular rearrangement induced by mechanical stresses; it is also responsible for the loss of energy during loading. Hysteresis is thus observed under loading cycles. The molecules’ rearrangement accommodates the stress and induces the appearance of a so-called back stress in the material; upon external load removal, the accumulated back stresses will induce the polymer to recover its original undeformed state. In this framework, the rubber-like response exhibited by viscoelastic materials is typically explained through the thermodynamic theory of elasticity applied to polymers. Their stress-strain rate response is usually categorized to be linear (Newtonian materials) or non-linear (non-Newtonian materials) according to the type of relationship existing between these two quantities. The mathematical modelling of viscoelastic phenomena is typically treated by rheological-based approaches, based on the classical Maxwell and Kelvin models whose mechanical response is obtained by connecting different dashpot/spring elements [18,19].

In the present paper, starting from a micromechanical perspective, we develop a model enabling the description of the viscous and failure behavior of polymers. The use of a statistical approach to describe the state of the polymer at the microscale is herein adopted; to this end, the so-called chains configuration space and the chain configuration density function are introduced and defined in Section 2. Such a function enables to have the complete knowledge of the state of the polymer and of its evolution in time. Once the above mentioned state is known—whose changes are triggered by the external mechanical actions, the viscous mechanism, and the failure of chains—it allows us to determine the deformation and the stress state of the material. In Section 3, the evolution of the chain density function is determined, while in Section 4 the proposed micromechanical model is discussed within a rigorous thermodynamic framework. After discussing the theoretical aspects of the model accounting for all the above mentioned mechanisms involved in polymers response, in Section 5 some parametric analyses, as well as mechanical tests taken from the literature, are simulated through the proposed approach and critically discussed. Some conclusions are finally drawn in Section 6.

## 2. Statistical Description of the Mechanics of Polymers

### 2.1. Chain-Based Models for Polymeric Materials

Polymers and polymer-like materials share a common feature: their microstructure is made of a complex network of entangled chains joined in several points called cross-links [20,21]; such cross-links can have a chemical nature (covalent bonds) [22,23] or they can be constituted by weaker physical bonds (such as ionic bonds) [24,25]. Several studies have considered the rheology of polymers; among them, it is worth mentioning one of the most successful models for the entangled polymer system, the so-called tube model proposed by Doi and Edwards [26].

The configuration of the chains arrangement for a given state of the material provides all the information required to completely describe such a state; the energy content, the deformation, and the stress state of the material can be determined once the chains conformation is known [8,11].

Several simplified models have been proposed in the literature to describe the mechanics of the chains network; generally, such models provide the elastic behavior of the network on the basis of simplified assumptions based on the spatial orientation of the chains. Within this class of models, it is worth mentioning the so-called 3-chains [27], the 4-chains (or Flory-Rehner) [4,28], the 8-chains [29], and the full network model [30].

For sake of exemplification, the simplest 3-chains model assumes the material to be made of several repeated identical unit cells, each one containing three freely jointed chains oriented along the three Cartesian axis; by assuming the affine deformation hypothesis, the free energy per unit volume of the material is given by $\Psi =\frac{1}{2}nk_{B}T\left(\lambda_{1}^{2}+\lambda_{2}^{2}+\lambda_{3}^{2}-3\right)$ (with $n,\;k_{B},\;T$ being the number of chains per unit volume, the Boltzmann constant and the absolute temperature, respectively, while $\lambda_{i},\;i=1,2,3$ are the macroscopic stretches existing along the Cartesian axes) that recovers the well-known neo-Hookean model according to which $\Psi =\frac{\mu }{2}\left(I_{1}-3\right)$, where $\mu =nk_{B}T$ is the shear moduls of the material, and $I_{1}$ is the trace of the Cauchy deformation tensor. More complex models (8-chains, full network, etc.) are simply based on more general similar assumptions.

### 2.2. The Chain Configuration Space and the Chain Configuration Density Function (CCDF)

By adopting the so-called freely jointed chain model (FJC), the state of a chain depends only on its end-to-end vector, i.e., on the vector r linking the chain’s ends. In the reference (stress-free) configuration state $\Omega_{0}$, the end-to-end chains’ vector is identified by $r_{0}$, while in a generic deformed state Ω such a vector is stretched to the new conformation given by $r=Fr_{0}$, being F the deformation gradient tensor describing the macroscopic deformation of the material. The above undeformed-deformed end-to-end vector relationship has been written according to the so-called affine deformation hypothesis [21]; according to such an assumption the deformation of the polymer chains is equal to the deformation of the continuum medium in which the chains are embedded.

According to the freely joined chain model, the force experienced by a single chain depends only on its end-to-end vector, so we can associate to each point of the configuration space $\Omega =\left\{r|r\in {\mathbb{R}}^{3}\right\}$ to the corresponding chain force, i.e., $f:\Omega \to {\mathbb{R}}^{3}$, with

(1) $$f=\frac{d\psi }{dr}=f\left(r\right)\;\bar{r}$$

where $\psi \;\mathrm{and}\;\bar{r}$ are the free energy of the chain and the unit vector of r, respectively.

**Figure 1:**
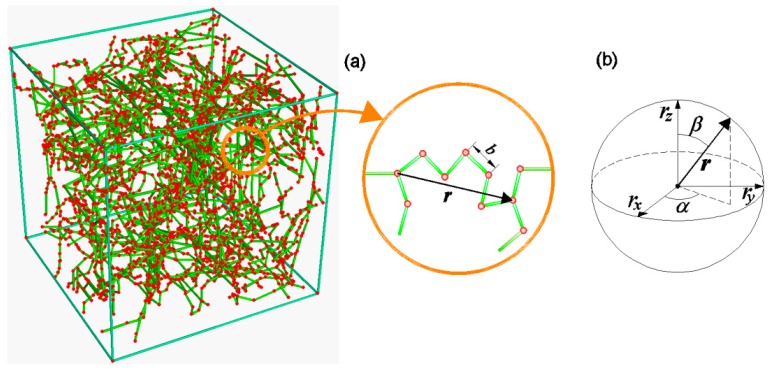
(**a**) Scheme of the polymeric chains network. (**b**) Definition of the end-to-end vector for a single chain in the configuration space $\Omega =\left\{\left(r_{x},r_{y},r_{z}\right)\in {\mathbb{R}}^{3}\right\}$.

Since the state of the polymer is completely defined through the conformation of its chains, it is convenient to introduce a scalar function ρ(r) providing the probability density of the chains distribution, i.e., the chain density (number of chains per unit volume of material) having a given end-to-end vector r (Figure 1). The above mentioned function can be identified as the Chain Configuration Density Function (CCDF), whose physical dimension is $\left[L^{-6}\right]$ (number of chains per unit volume of the configuration space and per unit volume of the physical space). It is convenient to write the CCDF function as:

(2) $$\rho \left(r\right)=c_{a}\cdot \varphi \left(r\right)$$

where $c_{a}$ is the chain density in the physical space (density of cross-linked chains), while φ(r) is the distribution function of r in the configuration space (accordingly, $\varphi_{0}\left(r\right)$ is the corresponding function in the undeformed state). It is worth noting that, since we are interested in the mechanical response of the polymer, only the bearing (active) chains have to be considered, i.e., those connected at both their extremities to others chains, while the free or dangling chains are not considered; in fact, because of the lack of connection to the network, their contribution to the material’s bearing mechanism is negligible. In this perspective, $c_{a}$ represents the density of the active chains only. The integral of ρ(r) over the configuration space provides the actual chains density, i.e.,

(3) $$\langle \rho \left(r\right)\rangle =\int_{\Omega }\rho \left(r\right)d\Omega =\int_{0}^{2\pi }\int_{0}^{\pi }\left(\int_{0}^{Nb}\rho \left(r\right)r^{2}dr\right)\sin\alpha d\alpha d\beta$$

where the last expression corresponds to the integration over the whole configuration space in spherical coordinates (r,α,β). It is worth noting that, being φ a probability density function, it must fulfill the condition 〈φ〉=1 [11].

Once the chains distribution is known, the energy per unit volume of material can be easily obtained as:

(4) $$\bar{\Psi }=\langle \rho \left(r\right)\;\psi \rangle$$

The above energy is greater than zero in the initial stress-free state, i.e., ${\bar{\Psi }}_{0}>0$ since it is ρ(r)≥0 ∀r∈Ω; the deformation energy Ψ associated to the deformed material has to be written as:

(5) $$\Psi =\bar{\Psi }-{\bar{\Psi }}_{0}=\langle [\rho \left(r\right)-\rho_{0}\left(r\right)]\;\psi \rangle$$

According to the above definition, the deformation energy Ψ becomes zero in the undeformed state, i.e., Ψ(F=I)=0 and $\rho \left(F=I\right)=\rho_{0}$.

From the above discussion, it appears that the mechanical state of the material is completely known once the function ρ(r) is provided, so the knowledge of its evolution—accounting for all the phenomena occurring in the material (deformation, chains sliding, etc.)—is sufficient to univocally determine the mechanical state of the material.

## 3. Modelling of the Visco-Elastic and the Damage Mechanisms of Polymers

In the present section, we develop a micromechanics-based model aimed at describing the visco-elastic and the damage response of polymers upon mechanical loading. Being the state of the material fully described once the Chain Configuration Density Function is known, its evolution, due to the various micromechanics phenomena involved, allows us to fully determine the mechanical state of the polymer, such as the stress and the damage level.

### 3.1. Microscale Approach: Evolution of the Chain Configuration Density Function

The evolution of the Chain Configuration Density Function (CCDF) in time (hereafter the time can be assumed to represent both the physical time or simply an increasing dimensionless quantity connected to the deformation) provides all the information needed to univocally define the state of the material.

The time derivative of the CCDF function can be decomposed as follows:

(6) $$\dot{\rho }={\dot{\rho }}_{L}+{\dot{\rho }}_{v}+{\dot{\rho }}_{f}$$

where ${\dot{\rho }}_{L}$ indicates the variation of ρ induced by the deformation (here $L=\dot{F}\;F^{-1}$ is the velocity deformation gradient), ${\dot{\rho }}_{v}$ is the contribution due to the viscous phenomena, and ${\dot{\rho }}_{f}$ provides the contribution of the chains failure to be accounted for if the material’s damage is assumed to occur. In the following, the three above contributions to the evolution of the CCDF function are singularly determined.

**Figure 2:**
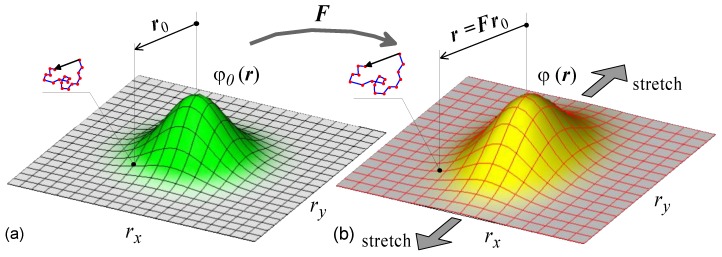
2-D scheme of the end-to-end distribution function in the undeformed state (**a**) and in the deformed one (**b**). The evolution, due to the deformation, from $\varphi_{0}\left(r\right)$ to a generic φ(r) obeys Equation (7).

The first contribution can be evaluated by considering the material behaving elastically; this implies that the number of connected (or active) chains in Ω does not change in time, since no chains are lost (damage) or gained (self-healing) during the deformation process. As a consequence, the time derivative of the chain concentration must vanish at any time, i.e., $\frac{d}{dt}\int_{\Omega }^{}\rho \;d\Omega =0$; consequently, it can be shown that the CCDF rate, due to the deformation of the material, in the elastic regime is expressed by [11].

(7) $${\dot{\rho }}_{L}=-\rho_{,i}\;{\dot{r}}_{,i}-\rho \;{\dot{r}}_{i,i}=-\left(\rho_{,i}\;r_{j}+\rho \;\delta_{ij}\right)L_{ij}$$

where ${\dot{r}}_{i}=L_{ij}r_{j}$ and ${\dot{r}}_{i,i}=L_{ii}$. It’s worth noting that the latter term in (7) ($\rho L_{ii}$) is zero for an incompressible material since the divergence of $\dot{r}$ vanishing for an isochoric deformation (i.e., $trL=L_{ii}=0$), so Equation (7) becomes ${\dot{\rho }}_{L}=-\rho_{,i}r_{j}\;L_{ij}$ or, in tensorial form, ${\dot{\rho }}_{L}=-\left(\nabla \rho ⊗r\right):L$ (Figure 2).

The second term in (6) refers to the time-dependent response of the material, so it must account for the internal microscopic rearrangement responsible for the viscous behavior. A simple model for such an internal material’s reorganization can be formulated by considering the network nature of a polymer: due to the fluctuating energy at the material microscale, the chains’ bonds can be assumed to detach from their stretched state and to reform again in the stress-free state of the material, i.e., after detaching they start being available again to contribute to the mechanical response [31,32]. From a statistical viewpoint, after the detachment, the chains reattach in the configuration $\varphi_{0}\left(r\right)$, corresponding to the initial macroscopically undeformed state of the material. In other words, the current number of cross-linked (i.e., active) chains arises from the dynamic balance between the attachment and the detachment process; this balance can be quantified once the detachment ($k_{d}$) and attachment ($k_{a}$) rates of the material are known [33]. These rates, in the simplest case, can be considered to be material’s parameter, even if it has been observed that they should depend on the intensity of the chain force; in particular, the force effect on $k_{d}$ can be described through the Kramer’s reaction rate theory, predicting an increasing of the detachment rate with the force increasing [34].

Once the polymerization of the material had occurred, the detachment/reattachment phenomenon reaches the steady state after a sufficiently long time and, in absence of damage or healing, the number of active chains does not change any more. The small deformation elastic shear modulus of the material can thus be associated to the steady state number of active chains. According to the 3-chains model, for instance, the chain concentration-shear modulus relationship is $c_{\mu }=\frac{\mu }{k_{B}T}$, with $c_{\mu }<c_{max}$, $c_{max}$ being the maximum potential value of active chains, i.e., the sum of all the attached and the detached ones at a given time instant. The evolution equation for $c_{\mu }$ can be written as [11,12]:

(8) $$\frac{dc_{a}\left(t\right)}{dt}=k_{a}\left(c_{max}-c_{a}\left(t\right)\right)-k_{d}c_{a}\left(t\right)$$

where the difference $c_{max}-c_{a}\left(t\right)$ represents the actual concentration of detached chains, while $k_{a}\;\mathrm{and}\;k_{d}$ are the activation and deactivation cross-link rates of the polymer chains, respectively. It can be easily verified that the steady state solution of Equation (8) is given by: $c_{a}\left(t\to \infty \right)=\frac{k_{a}}{k_{a}+k_{d}}c_{max}=c_{\mu }$, where the last equality is justified because the shear modulus is assumed to be measured in the steady-state condition. By considering that the active chains detach from the actual (deformed) distribution φ(r) and the ones that become active begin to be stretched starting from the initial (reference) one, $\varphi_{0}\left(r\right)$, the density distribution rate due to the material microstructure rearrangement is expressed as:

(9) $${\dot{\varphi }}_{v}\left(t\right)=k_{a}\frac{c_{max}-c_{a}\left(t\right)}{c_{a}}\varphi_{0}-k_{d}\varphi \left(t\right)$$

while the corresponding CCDF evolution, evaluated at constant applied deformation, assumes the form:

(10) $${\dot{\rho }}_{v}\left(t\right)=k_{a}\left[c_{max}-c_{a}\left(t\right)\right]\varphi_{0}-k_{d}\rho \left(t\right)=-k_{d}c_{\mu }\left(\varphi \left(t\right)-\varphi_{0}\right)$$

where chains conservation at the steady state, $dc_{a}/dt=0$, has been leveraged.

It is reasonable to assume that, when the polymer is formed, i.e., when the polymerization process is complete, the concentration of active chains corresponds to that in the steady state situation, so $c_{a}\left(t=0\right)=c_{a0}=c_{\mu }$. Finally, it is worth mentioning that the above mentioned parameters $k_{a}\;\mathrm{and}\;k_{d}$ can be easily related to the well-known loss and storage modulus, typically used in rheology to characterize the material’s viscous behavior [11].

Finally, we consider the CCDF evolution due to the chain scission. The loss of chains entails a modification of the previous chains conservation equation, i.e., now $dc_{a}/dt\leq 0$; the general expression of the CCDF evolution provided by Equation (6) must now be updated accordingly:

(11) $$\dot{\rho }=-\mathrm{div}\left(\rho \dot{r}\right)+{\dot{\rho }}_{f}$$

where the second term on the right hand side represents the rate of loss chains in the configuration space. The explicit expression for ${\dot{\rho }}_{f}$ can be obtained by introducing the failure rate function $\omega_{f}\left(r\right)$ that provides the fraction of broken chains per unit time for a given chain’s end-to-end vector r; the CCDF rate due to the chains failure is then

(12) $${\dot{\rho }}_{f}=-\omega_{f}\left(r\right)\;\rho \left(r\right)$$

This indicates that the number of chains lost per unit time is proportional through the function $\omega_{f}$; the actual number of active chains for a given end-to-end vector r is quantified by the CCDF ρ(r).

The above expression implies a loss of material or, equivalently, of active chains; this entails that the material undergoes a permanent damage because of the chain loss; it is thus possible to define a scalar damage parameter of the material at the current time instant to be defined as:

(13) D(t)=1−〈φ(t)〉, 0≤D(t)≤1

It happens to be D(t)=0 for an undamaged polymer (i.e., 〈φ(t)〉=1), while D(t)=1 for a fully damaged material; the latter case corresponds to $c_{a}\left(t\right)=c_{\mu }\langle \varphi \left(t\right)\rangle =0$ because no active chains exist anymore if the material is fully broken.

The failure rate function $\omega_{f}\left(r\right)$ is the key factor to quantify the phenomenon of permanent chain loss; in this context, the chains’ bond failure represents the physical phenomenon responsible for the chains break. It is reasonable to describe such an irreversible process by adopting the well-known Eyring’s reaction rate theory [35]. For the family of polymer’s chains having the end-to-end vector r, the failure rate can be expressed by [36]:

(14) $$\omega_{f}\left(r\right)=H\left(r\cdot \dot{r}\right)\frac{1}{\tau }G\left(w_{b}\left(r\right)\right),\;\mathrm{with}\;H\left(r\cdot \dot{r}\right)=\{\begin{array}{c} 1\;if\;r\cdot \dot{r}>0\\ 0\;if\;r\cdot \dot{r}\leq 0 \end{array}$$

In the above expression τ represents a characteristic time, while $w_{b}$ is the chain’s segments deformation energy available to induce the chain’s bond failure. The function 0≤G(w(r))≤1, having the unit of $t^{-1}$, provides the probability of failure per single chain’s segment and per unit time, i.e., the fraction (over the existing chains of a given length) of chain loss (or chain scission) per unit time. By introducing the chain’s bond strength energy $\bar{w}$, the above equation can be rewritten as follows:

(15) $$\omega_{f}\left(r\right)=H\left(r\cdot \dot{r}\right)\cdot {\tilde{\omega }}_{f}\left(r\right)=H\left(r\cdot \dot{r}\right)\cdot A\frac{k_{B}T}{h}\left[\frac{1}{1+\exp\left(\gamma \frac{w_{b}-\bar{w}}{k_{B}T}\right)}\right]\geq 0$$

where A is a model parameter, $w_{b}=\frac{1}{2}E_{b}\ln^{2}\lambda_{b}$ is the (enthalpic) deformation energy of one chain’s Kuhn segment [37] ($E_{b},\;\lambda_{b}$ being the stiffness and the stretch of the Kuhn segments, respectively, where $\lambda_{b}$ can be determined from the stationarity condition of the sum of the entropic and the enthalpic parts of the chain energy of the material, [9]), h is the Planck’s constant, and γ<0 is a parameter whose value governs the sharpness of the transition between unfailed ($w_{b}<\bar{w}$) and failed state ($w_{b}\geq \bar{w}$) of the chains. The Heaviside step function $H\left(r\cdot \dot{r}\right)$ has been introduced in (14) in order to distinguish between the state of loading or unloading for a single chain; during loading the chain increases its length ($r\cdot \dot{r}>0$), and the damage can take place in the material (H=1). During unloading the chain shortens ($r\cdot \dot{r}\leq 0$), and its damage cannot increase (H=0).

**Figure 3:**
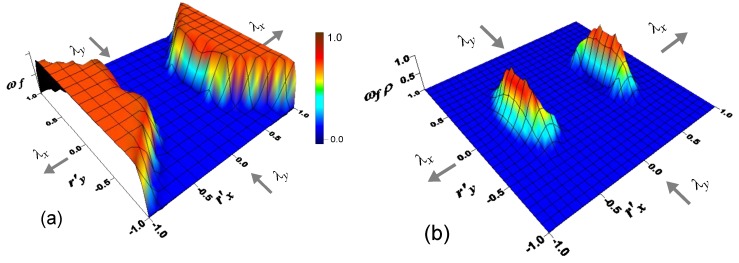

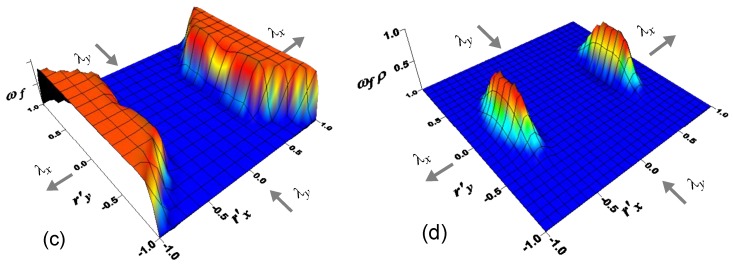
Contour plot surface of the failure rate $\omega_{f}$ in the $r_{x},\;r_{y}$ space for a bond strength $\bar{w}$ (**a**) and corresponding Chain Configuration Density (CCDF) rate $\omega_{f}\left(r\right)\;\rho \left(r\right)$ due to chains failure (**b**). In (**c**,**d**), the same graphs are shown for a bond strength 2$\bar{w}$.

The graphical representation in the 2-D space $r_{x},\;r_{y}$ (the dimensionless quantities ${r^{’}}_{x}=\frac{r_{x}}{b\sqrt{N}},\;{r^{\prime }}_{y}=r_{y}/\left(b\sqrt{N}\right)$ have been used for sake of simplicity) of Equation (15) is provided in Figure 3a,c for a deformation process taking place in plane stress condition characterized by $\lambda_{y}=\lambda_{z}=\lambda_{x}^{-1/2}$, where the failure rate $\omega_{f}$ is illustrated (Figure 3b,d) for a polymer for two assumed values of the bond energy $\bar{w}$.

The CCDF rate $\omega_{f}\rho$ due to the chains failure is depicted in Figure 3b,d; the major portion of the lost chains (see peaks of Figure 3b,d) corresponds to the chains that lie mainly aligned along the stretch direction x and has a sufficiently large end-to-end vector length, i.e., for those chains with a deformation energy closed to the bonding one, $w_{b}\geq \bar{w}$. Shorter chains do not fail because they are not enough stretched, while chains longer than the failed ones do not practically exist in the network; it’s worth noting that the chains lying along directions mainly elongated in the y-direction do not fail either because they are subjected to unloading for the adopted deformation process.

An approach for polymer damage based on the probability of failure in the chain configuration space has been recently proposed in [38]; the present approach differs from the above mentioned one being based on the kinetic of the chain failure through the reaction rate theory, whose main parameter is the chain dissociation energy. On the other hand, the statistical damage approach in [38] assumes an initial failure distribution function in the chains’ configuration space and makes it to evolve according to the damage occurred in the material. The problem of polymer’s chains failure ahead of a crack in a soft rubbery material has been considered by Hui et al. [39]: they adopted a cohesive model characterized by a failure force whose value depends on the thermal state of the material and on the rate at which the force is transmitted to the bond (thermo-mechanically activated bond dissociation kinetics).

### 3.2. Stress State in the Polymer

As mentioned before, the knowledge of the distribution function φ enables to completely know the mechanical state of the material; in particular, the stress state can be easily obtained as $P\left(t\right)=\frac{\partial \Psi \left(t\right)}{\partial F}+p\left(t\right)JF^{-T}$, with P and p being the first Piola stress tensor and the hydrostatic pressure enforcing the isochoric deformation, respectively, and J=detF being the volumetric deformation. On the other hand, the Cauchy stress is provided by the relation $\sigma =J^{-1}PF^{T}$ and can be demonstrated to be obtainable from the relationship [40]:

(16) $$\begin{array}{cc} \sigma \left(t\right)=J^{-1}\left(\frac{\partial \Psi }{\partial t}\frac{\partial t}{\partial F}\right)F^{T} & =c_{a}\int_{\Omega }\left[\varphi \left(t\right)-\varphi_{0}\left(t\right)\right]f\left(t\right)⊗r\;d\Omega +p\left(t\right)I=\\ & =\int_{\Omega }\left[\rho \left(t\right)-\rho_{0}\left(t\right)\right]f\left(t\right)⊗rd\Omega +p\left(t\right)I \end{array}$$

where it has been assumed J=detF=1 (due to the incompressibility constraint), while f is the force existing in the chains of a given end-to-end distance r (evaluable by a suitable model such as through the well-known Langevin statistics, $f=\frac{\partial \psi \left(t\right)}{\partial r}=\frac{1}{b\sqrt{N}}\frac{\partial \psi }{\partial \lambda }=\frac{r}{\left|r\right|}\cdot \frac{k_{B}T}{b}\cdot L^{-1}\left(\frac{\lambda }{\sqrt{N}}\right)$). It’s worth noting that the chain density distribution function in the reference configuration $\rho_{0}\left(t\right)$ has to be updated in the case the damage occurs in the material, and so the time dependence of $\rho_{0}$ has been explicitly indicated in (16). In fact, in this case the stress-free chains’ configuration corresponds to the current chain density distribution function ρ(t) brought back to the un-deformed state, i.e., for F=I. In other words, the current distribution function in the reference state, $\varphi_{0}\left(t\right)$, must correspond to φ(t), being $\langle \varphi_{0}\left(t\right)\rangle =\langle \varphi \left(t\right)\rangle \leq \langle \varphi \left(t=0\right)\rangle =1$ due to the chains loss; correspondingly, the density of the actual active chains is now $c_{a}\left(t\right)\leq c_{a}\left(t=0\right)$. The pull-back operation is provided by the following relation

(17) $$\varphi_{0}\left(r,t\right)=\varphi \left(Fr,t\right),$$

i.e., at the current time instant t the value of the reference distribution function $\varphi_{0}$ for a given r must correspond to the value assumed by φ for the end-to-end vector Fr. The above described pull back operation is unnecessary if the damage is not occurring in the material, and in these cases it is simply $\varphi_{0}\left(r,t\right)=\varphi_{0}\left(r,t=0\right)$.

### 3.3. Polymers with Multiple Networks

The above presented micromechanical model has been developed under the hypothesis that the polymer is made of a single network, i.e., all the chains are made of a fixed number N of Kuhn’s segments. However, in real polymers the presence of chains composed by different numbers of segments can be found (these polymers also termed as polydisperse networks or networks with a non-uniform weight distribution) [41,42]. Within this context, the development of polymers with double networks has attracted a lot of efforts in recent years because of their capability to dissipate energy; thanks to the sacrificial role that can be attributed to the more brittle network, the more flexible ones can act as a bridging skeleton, enhancing the fracture resistance of the polymer [42,43]. Theoretical approaches have been also developed to describe the mechanics of polydisperse networks [44,45].

The proposed model can be easily extended to the case of polydisperse networks by introducing the distribution function of the Kuhn’s segments number, q(N); accordingly, the energy per unit volume (5) has to be updated as follows:

(18) $$\Psi =\int_{N_{min}}^{N_{max}}q\left(N\right)\;\langle [\rho \left(N,r\right)-\rho_{0}\left(N,r\right)]\;\psi \rangle \;dN$$

where $N_{min}$ and $N_{max}$ represent the number of segments of the shortest and of the longest network in the polymer, respectively; in the case of a finite number m of different networks, the above expression must be replaced by a summation over all the networks, being $\sum_{N_{i}=N_{1}}^{N_{m}}q\left(N_{i}\right)=1$, while $q\left(N_{i}\right)$ represents the volume fracion of the i-th network. In this case, the evolution of the CCDF (Equation (6)) has to be evaluated with reference to each single network, i.e., for a specific value of $N_{min}\leq N\leq N_{max},\;\dot{\rho }\left(N,r\right)={\dot{\rho }}_{L}\left(N,r\right)+{\dot{\rho }}_{v}\left(N,r\right)+{\dot{\rho }}_{f}\left(N,r\right)$, where the contributions of the deformation (7), of the viscous mechanism (10), and of the chains failure (12) have to be evaluated according to the deformation and the stress state acting on each single network.

## 4. Thermodynamics of Polymers Undergoing Chains Failure and Bond Exchange

From an energetic viewpoint, the problem under consideration must comply the first principle of thermodynamics; it implies that the material derivative of the internal energy density $E_{n}$ (per unit current volume) can be expressed by:

(19) $$\dot{E_{n}}=\sigma :L-\nabla \cdot q+s$$

where σ is the Cauchy stress tensor, q the heat flux in a given point, and s is the rate of external heat supply per unit current volume. On the other hand, the Clausius-Duhem inequality provides the second principle of thermodynamics:

(20) $$\dot{\vartheta }\geq \frac{s}{T}-\nabla \cdot \left(\frac{q}{T}\right)$$

with ϑ being the entropy per unit current volume and T the absolute temperature. By introducing the Helmholtz free energy per unit current volume, $\Xi =E_{n}-T\vartheta$, the second principle becomes:

(21) $$\sigma :L-\dot{\Xi }-\dot{T}\vartheta -\frac{q}{T}\cdot \nabla T\geq 0$$

For sake of convenience, let us assume an incompressible polymer, and postulate that the deformation process takes place isothermally ($\dot{T}=0$) and without any external heat supplied (q=0, s=0), so $\sigma :L-\dot{\Xi }\geq 0$. In this particular case, the rate of change of the Helmholtz free energy per unit current volume becomes:

(22) $$\dot{\Xi }=\dot{\Psi }=\int_{\Omega }\dot{\rho }\left(r,t\right)\psi \;d\Omega =\int_{\Omega }\left[\left(\frac{\partial \rho \left(r,t\right)}{\partial r}r+\nabla \cdot \left(\rho \left(r,t\right)L\cdot r\right)\right):L\right]\psi \;d\Omega -D=\sigma :L-D$$

where D represents the dissipated energy. By replacing (7), (10), and (12) in (22), we can recognize the energy loss to be expressed by:

(23) $$D\left(t\right)=\int_{\Omega }\left[-k_{d}c_{\mu }\left(\varphi \left(t\right)-\varphi_{0}\right)+H\left(r\cdot \dot{r}\right)\cdot {\tilde{\omega }}_{f}\left(r\right)\;\rho \right]\psi \;d\Omega ,$$

i.e., it is provided by the contributions coming from the time-dependent response of the material and the one from the chains failure. Being the viscous and the damage contributions independent of each other, at any time instant t, the positiveness of the dissipated energy

(24) $$D\left(t\right)=D_{v}\left(t\right)+D_{f}\left(t\right)\geq 0,\;\forall t$$

is ensured if both the following conditions hold:

(25) $$D_{v}\left(t\right)=\int_{\Omega }\left[-k_{d}c_{\mu }\left(\varphi \left(t\right)-\varphi_{0}\right)\right]\psi \;d\Omega \geq 0\;D_{f}\left(t\right)=\int_{\Omega }H\left(r\cdot \dot{r}\right)\cdot {\tilde{\omega }}_{f}\left(r\right)\;\rho \left(r,t\right)\;\psi \;d\Omega \geq 0$$

The first condition is fulfilled if $\int_{\Omega }\left[\left(\varphi \left(t\right)-\varphi_{0}\right)\right]\psi \;d\Omega \leq 0$; it corresponds to the non-increasing character of the energy stored in the polymer, i.e., the inequality $\int_{\Omega }\varphi \left(t\right)\psi \;d\Omega \leq \int_{\Omega }\varphi_{0}\psi \;d\Omega$ guarantees that, because of the network relaxation, the chains detachment is associated with a decrease in the stored elastic energy and that this loss is proportional to the rate of dissociation $k_{d}$ and the stored elastic energy, $\int_{\Omega }\left[c_{\mu }\left(\varphi \left(t\right)-\varphi_{0}\right)\right]\psi \;d\Omega$.

The second requirement in (25) is consistent with (15), since ${\tilde{\omega }}_{f},\;\rho ,\;\psi$ are all positive functions ∀r, while, according to (14), $H\left(r\cdot \dot{r}\right)$ is positive when the chain undergoes loading, $r\cdot \dot{r}>0$ (i.e., increase of its end-to-end vector); meanwhile, it becomes zero in the case of neutral loading, $r\cdot \dot{r}=0$, or unloading, i.e., $r\cdot \dot{r}<0$ (no change or decrease of its end-to-end vector).

## 5. Application of the Micromechanical Model

In the following some parametric tests as well as simulations of real polymer tests are presented in order to underline the main features of the proposed model for the viscous and damage response of polymers.

### 5.1. Parametric Analyses

In the present section, we present some parametric tests aimed at underlying the role played by the main parameters involved in the above presented time-dependent and damage model to quantify the response of polymers under mechanical actions. Firstly, the rate dependent response is analyzed.

#### 5.1.1. Rate-Dependent Response

In this case, the time-dependent response, in absence of any damage phenomena, is modelled through the chains attachment-detachment mechanism described in Section 3.1, leading to the evolution in time of the network microstructure (see Equation (10)).

In particular, we consider the analysis of a polymer under two stretching cycles (in each one the stretch starts from the un-deformed state, λ=1 to the deformed one, λ=2, and, afterwards, the stretch goes back to λ=1). The material is assumed to be incompressible and characterized by an elastic and a shear modulus, E=5 MPa, μ=1.67 MPa, T=300 K, and N=50, while the rate parameters $k_{a}\;\mathrm{and}\;k_{d}$ are made to vary as follows: $k_{a}=1\;\mathrm{Hz}$ (kept constant), (i) $k_{d}=0\;\mathrm{Hz}$, (ii) $k_{d}=0.1\;\mathrm{Hz}$, and (iii) $k_{d}=0.3\;\mathrm{Hz}$. Finally, two strain rates have been adopted, i.e., the slowest case with $\dot{\lambda }=0.2\;s^{-1}$ and the fastest one characterized by $\dot{\lambda }=2\;s^{-1}$.

**Figure 4:**
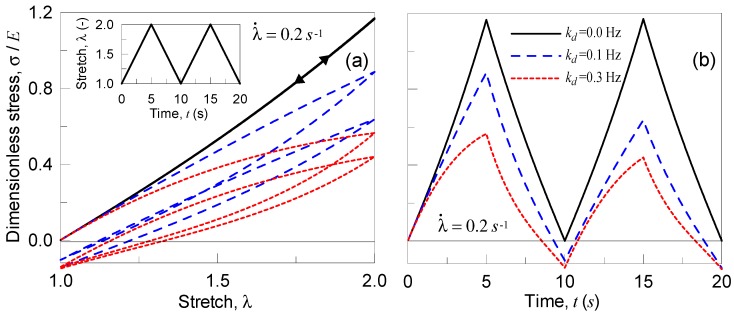
Dimensionless Cauchy stress vs stretch (**a**) and vs time (**b**) for the three examined polymers characterized by different deactivation cross-link rates for a deformation rate of $\dot{\lambda }=0.2\;s^{-1}$.

In Figure 4, the results obtained for the case of slow strain rate, $\dot{\lambda }=0.2\;s^{-1}$, are reported in terms of dimensionless true stress vs deformation (Figure 4a) and vs time (Figure 4b). Greater $k_{d}$ values lead to a greater dissipation (wider hysteresis loops), while, correspondingly, the maximum stress decreases and the stress state after unloading results to be negative; this latter observation indicates that the material has to be compressed in order to be brought to its original un-deformed state, because of the internal resetting occurred during the deformation process. Such an internal resetting is much more pronounced for higher values of $k_{d}$.

**Figure 5:**
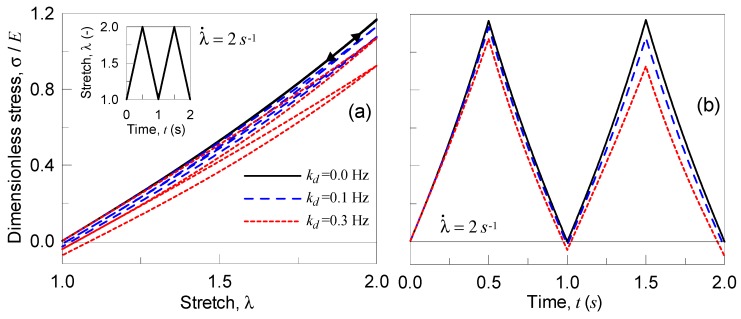
Dimensionless Cauchy stress vs stretch (**a**) and vs time (**b**) for the three examined polymers characterized by different deactivation cross-link rates for a deformation rate of $\dot{\lambda }=2\;s^{-1}$.

By adopting a faster strain rate value, $\dot{\lambda }=2\;s^{-1}$, the material responds as indicated in Figure 5; the strain rate is greater than in the first examined case, for the same rate parameters $k_{a},\;k_{d}$ the material has not enough time to reset internally its network, so the behavior is more close to the elastic one.

**Figure 6:**
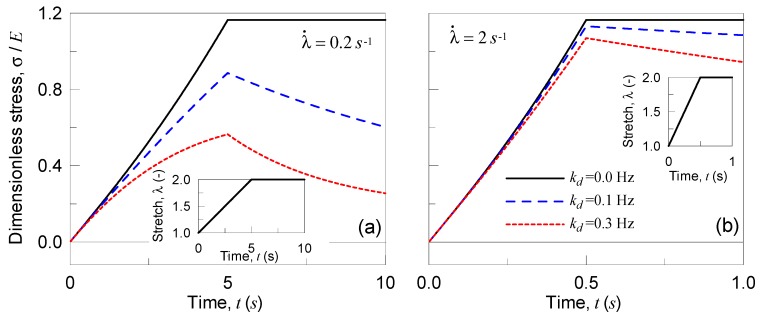
Dimensionless Cauchy stress vs time for three different values of the detaching rate $k_{d}$. During the loading ramp the stretch rate has been assumed to be equal to 0.2 s^−1^ (**a**) and to 2 s^−1^ (**b**).

In the second parametric test, the material is stretched to a given value, and then the deformation is kept constant (see details in Figure 6); the applied deformation is quantified through the parameter 0≤α(t)≤1.0, being $\lambda \left(t\right)=\lambda_{0}\cdot \alpha \left(t\right)$ with $\lambda_{0}=2$. It can be observed that the material behaves perfectly linearly when no chains detachment occurs ($k_{d}=0$); meanwhile, by increasing the detachment frequency, the stress reached in the material for a given stretch decreases (Figure 6). Moreover, by keeping the deformation constant, the material relaxes itself more significantly for higher $k_{d}$ values; finally, the elastic case is recovered in the particular case $k_{d}=0$.

#### 5.1.2. Mechanical Response of a Single Network Polymer in Presence of Damage

**Figure 7:**
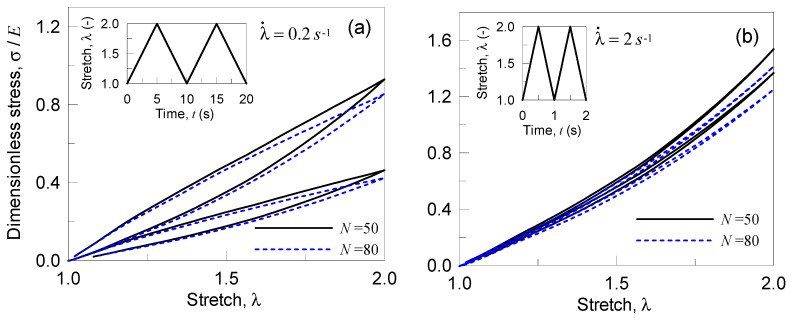
Stress-stretch curves for a single network polymer (with N=50 or N=80) in presence of damage: response under the stretch rate rate $\dot{\lambda }=0.2\;s^{-1}$ (**a**) and $\dot{\lambda }=2\;s^{-1}$ (**b**). The bond energy strength has been assumed to be $\bar{w}=33.82\;k_{B}T$, while the Kuhn segment stiffness parameter has been adopted to be $E_{b}=2300\;k_{B}T$.

**Figure 8:**
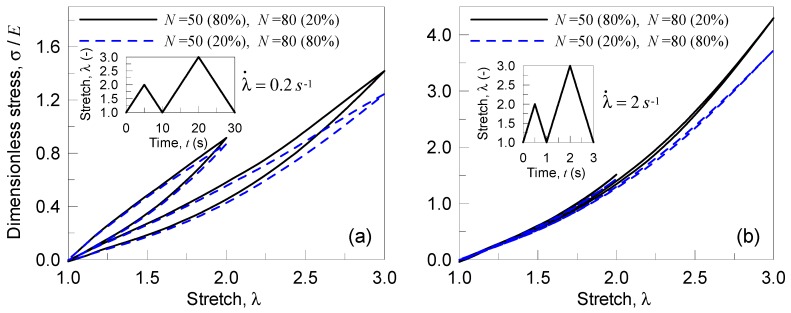
Stress-stretch curves for a double network polymer (with $N_{1}=50$ and $N_{2}=80$ according to the percentages indicated in the legends) in presence of damage: response under two stretch cycles (characterized by a stretch rate rate $\dot{\lambda }=0.2\;s^{-1}$ (**a**) and $\dot{\lambda }=2\;s^{-1}$ (**b**)) with increasing amplitude. The bond energy strength $\bar{w}=33.82\;k_{B}T$ has been assumed, while the Kuhn segment stiffness parameter has been adopted to be $E_{b}=2300\;k_{B}T$.

In the second case, we analyze the response of a single network polymer by accounting for the occurrence of damage without any internal rearrangement features ($k_{d}=0\;\mathrm{Hz}$). The CCDF time evolution, $\dot{\rho_{f}}$, is here evaluated according to Equation (12), while the failure rate function $\omega_{f}\left(r\right)$ is defined according to Equation (15). The material’s parameters are the following: Elastic modulus E=20 MPa, shear modulus μ=6.67 MPa, N=50 or N=80, and T=300 K, while the bond energy has been adopted to be $\bar{w}=33.82\;k_{B}T\;\left(=1.4\cdot 10^{-19}\;J\right)$, $E_{b}=2300\;k_{B}T$ [9], and A=0.012, γ=−0.8. The material is subjected to two deformation cycles with 1≤λ(t)≤2 characterized by strain rates equal to $\dot{\lambda }=0.2\;s^{-1}$ and $\dot{\lambda }=2\;s^{-1}$. In Figure 7, the stress-deformation response is illustrated for the two different networks considered and for the two strain rates adopted; it can be noted that the polymer with longer chains (N=80) responds more softly, and that in the high strain rate case the damage is lower because of the short time available to the chains to break during the deformation process (Figure 8b).

#### 5.1.3. Mechanical Response of a Polydisperse Polymer in Presence of Damage

In this case, we consider the response of a polydisperse polymer; the material’s characteristics have been assumed the same as those presented in the previous section, while now the material is assumed to be made of two networks, one with $N_{1}=50$ and the second with $N_{2}=80$. The volume fractions of the two networks have been assumed to be $q\left(N_{1}\right)=0.8$, $q\left(N_{2}\right)=0.2$ for the first polydisperse polymer and $q\left(N_{1}\right)=0.2$, $q\left(N_{2}\right)=0.8$ for the second multi-networks polymer. The material is subjected to two deformation cycles, with 1≤λ(t)≤2 in the first cycle and 1≤λ(t)≤3 in the second one; each of the deformation history has been assumed to be characterized by the strain rates equal $\dot{\lambda }=0.2\;s^{-1}$ or $\dot{\lambda }=2\;s^{-1}$.

In Figure 8, the response of the material is shown for the two double networks polymers and for the two strain rates considered; the case of the polymer richest of the shortest chains ($N_{1}=50$) shows a stiffer response with respect to the one with $q\left(N_{2}\right)=0.8$. As in the previous parametric analyses, a high value of the strain rate implies a lower energy dissipation and the response is very close to the purely elastic case.

### 5.2. Simulations of Experimental Tests

**Figure 9:**
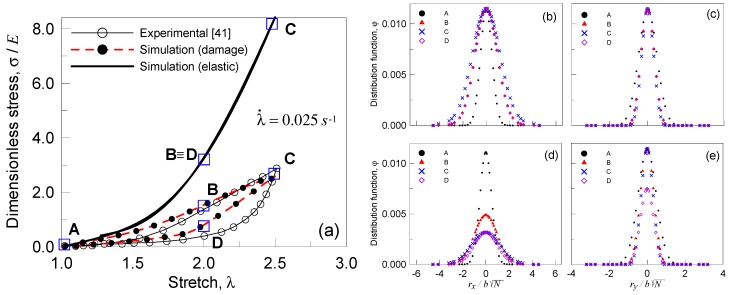
Dimensionless stress vs stretch curves for a triple network polymer (with $N_{1}=15$, $N_{2}=80$ and $N_{3}=160$) in presence of damage: response under two stretch cycles (characterized by a stretch rate $\dot{\lambda }=0.2\;s^{-1}$ (**a**) and $\dot{\lambda }=2\;s^{-1}$ (**b**)) with increasing amplitude (**a**). The bond energy strength $\bar{w}=33.82\;k_{B}T$ has been assumed, while the Kuhn segment stiffness parameter has been adopted to be $E_{b}=2300\;k_{B}T$. Cross-section of the distribution function φ(r) of the first network along the $r_{x}$ (**b**,**d**) and $r_{y}$ (**c**,**e**) axis at various steps (see the squares in (**a**)) of the deformation history. Case of purely elastic response (**b**,**c**) and response in presence of damage (**d**,**e**).

In the present section, we finally consider the simulation of experimental tests [46]. In order to get a satisfactory agreement with the experimental results, we identify that the optimal number of Kuhn segments in the chains of each network is $N_{1}=15$ ($q(N_{1})=0.8$), $N_{2}=80$ ($q(N_{2})=0.1$), and $N_{3}=160$ ($q(N_{3})=0.1$). A deformation cycle with 1≤λ≤2.5 with $\dot{\lambda }=0.025\;s^{-1}$ has been considered, while E=5 MPa, μ=1.67 MPa, and T=300 K. Moreover, with the same monomer used in each network, the damage parameters have been adopted to be as follows for all the three networks: $\bar{w}=33.82\;k_{B}T\;\left(=1.4\cdot 10^{-19}\;J\right)$, $E_{b}=2300\;k_{B}T$ [9], and A=0.0019, γ=−0.8. Moreover, the material is supposedly incompressible and to be stretched along the x-direction (parallel to the $r_{x}$ axis). The experimental response and the simulated one, for both the cases of elastic and damage behavior, are illustrated in Figure 9a; the usual stiffening behavior can be appreciated for the elastic case (to this end the damage rate function has been made to vanish, $\omega_{f}\left(r\right)=0$), while a considerable dissipation of energy can be appreciated for the response in presence of damage.

Further, the cross-section of the distribution function φ(r) related to the first network ($N_{1}=15$, the one present with the greatest volume fraction $q(N_{1})=0.8$) along the $r_{x}$ (Figure 9b,d) and $r_{y}$ (Figure 9c,e) axis evaluated at various steps of the deformation history has been determined. The deformation states corresponding to the points indicated with A, B, C, and D in Figure 9a have been considered for the elastic as well as for the damage response; it can be appreciated that such a distribution widens in the direction of the applied deformation (Figure 9b,d) and narrows in the normal direction. It can be noticed that the occurrence of the damage is reflected in the decreasing of the area under the distribution function (Figure 9d) due to the chains loss. On the other hand, the chains oriented in the direction normal to the applied stretch do not fail easily, as witnessed by the distribution function’s sections along the $r_{y}$ axis; the sections’ pattern related to the elastic response (Figure 9c) is very similar to that related to the behavior in presence of damage (Figure 9e).

## 6. Conclusions

The macroscopic mechanical response of polymers, usually characterized by a high nonlinearity degree, damage, and strain rate dependence, depends on many complex mechanisms taking place at the microscale. Among them, it is worth mentioning the existence of chains reversible bonds, bond exchange, chains sliding, chains’ bond scission, and chains uncoiling. In the present study, a theoretical micromechanical model enabling to study the time-dependent response of polymers by accounting for the damage mechanism taking place at the microscale has been proposed. The proposed approach has been developed by introducing the so-called Chain Configuration Density Function (CCDF), defined over the chains configuration space; starting from the network’s chain statistics, the evolution of such a function has been determined by accounting for the deformation, the rate-dependence and the damage mechanism. The developed approach has been up-scaled to the mesoscale by integrating the main field quantities over the above mentioned chains configuration space. The knowledge of the CCDF at a given instant of the deformation history provides all the necessary information required to fully know the mechanical state of the material.

The thermodynamic consistency of the theoretical framework has been illustrated, and its reliability in modelling the response of polymers has been illustrated through some parametric examples involving the rate dependent response of elastomers and the damage behavior under deformation cycles. An experimental test has also been simulated, and the corresponding CCDF evolution has been shown and discussed. The main advantage of the developed theoretical model can be acknowledged in its physics-based micromechanical nature; the main mechanisms involved at the scale of the polymer’s network reflect the macroscopic material’s mechanical characteristics, and the need for few parameters having a clear physical meaning (such as the shear modulus, the loss and storage modulus, and the cross-link bond strength) enable the approach to be easily used and implemented in computational code to predict the complex response of polymers.

## Nomenclature

A	Parameter of the damage rate function $\omega_{f}$
b	Length of a chain Kuhn’s segment
$c_{a},\;c_{max}$	Actual and maximum number of active chain per unit volume, respectively
D	Damage parameter of the material
D	Dissipated energy per unit volume of material
$D_{v},D_{f}$	Dissipated energy per unit volume of material related to the viscous and to the chain’s failure mechanisms, respectively
$F,F_{ij}$	Deformation gradient tensor
t	Time
J	Polymer volume change ratio
$k_{a},k_{d}$	Activation and deactivation cross-link rates of the polymer chains, respectively
$k_{B}$	Boltzmann’s constant
L, $L_{ij}$	Spatial velocity gradient
$L,\;L^{-1}$	Langevin function and its inverse, respectively
N	Number of segments in a polymer chain belonging to a single network
q(N)	Probability distribution function of the number of Kuhn’s segments in a polydisperse polymer
r	End-to-end vector of a polymer chain
f	Force in a single stretched chain
T	Absolute temperature
$w_{b}$	Strain energy of a single Kuhn segment
$\bar{w}$	Bond energy between two segments of a polymer chain
γ	Parameter of the damage rate function $\omega_{f}$
$\varphi_{0}\left(r\right),\varphi \left(r\right)$	Distribution function of the end-to-end vector in the stress-free and in the current state, respectively
λ	Unidimensional stretch measure
$\omega_{f}\left(r\right)$	Failure rate related to the chains with end-to-end vector r
ρ(r)	Chain Configuration Density Function (CCDF)
$\dot{\rho },\;{\dot{\rho }}_{L},\;{\dot{\rho }}_{v},\;{\dot{\rho }}_{f}$	Total CCDF rate, CCDF rate due to deformation, viscous effects and failure, respectively
ψ	Deformation energy for a single chain
$\Psi_{0},\Psi$	Network’s deformation energy per unit volume in the stress-free and in the current configuration, respectively
σ	Chauchy stress
